# Supplementary material for: Tailoring van der Waals dispersion interactions with external electric charges
Source: Nat Commun. 2018 Aug 1;9:3017. doi: 10.1038/s41467-018-05407-x (PMC6070553; doi:10.1038/s41467-018-05407-x)
Supplement: Supplementary file 1 — Supplementary Information [file 41467_2018_5407_MOESM1_ESM.pdf]

Supplementary information for Tailoring van der Waals Dispersion Interactions With External Electric Charges

Andrii Kleshchonok and Alexandre Tkatchenko

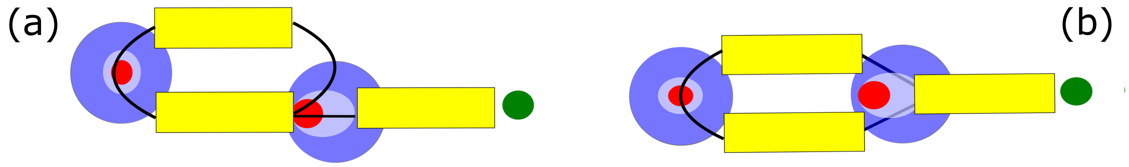

Supplementary Figure 1. Induced dipole-quadrupole dispersion (a) and electrostatic (b) terms in the diagrammatic representation. The QDOs are sketched by blue circles and the external charge is shown with a green dot.

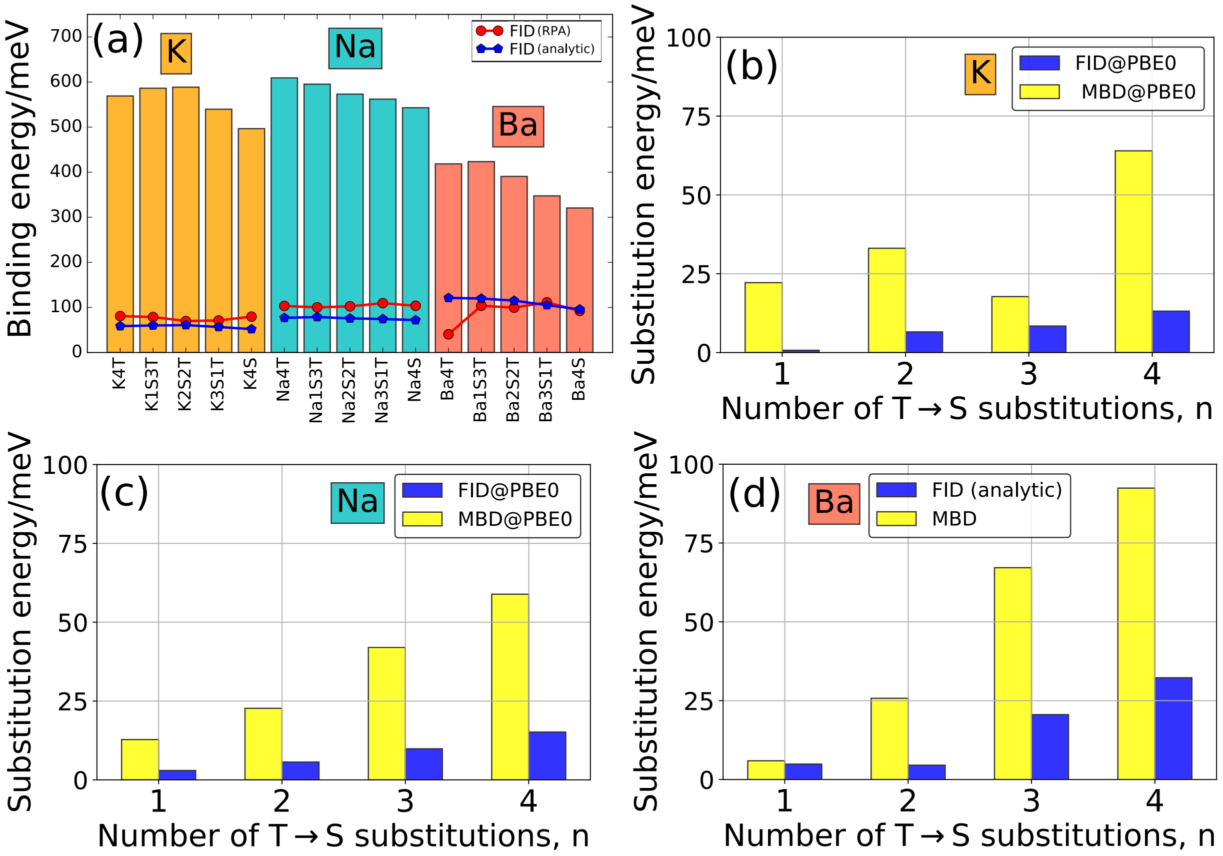

Supplementary Figure 2.(a) Absolute value of FID W_c^{bind} (in meV) obtained from RPA calculations (red line) and analytic Eq.15 (blue line) compared to the MBD for K^+ (orange bars), Na^+ (cyan nars), Ba^{2+} (pink bars). Substitutional MBD energies (shown in yellow) and substituitional FID (blue) contributions for K^+ (b), Na^+ (c) and Ba^{2+} (d) ion complexes.

Supplementary Table 1. First two columns show contribution of FID energy to the total binding energy between molecular dimers and the effect of an external charge on total DFT binding energy given by the PBE0 functional (third and forth columns), evaluating mostly electrostatic contributions. With "*" symbol we denote cases with 5 Å distance from dimer center of mass to the external charge and 3 Å otherwise.

Molecular dimer	$\Delta E(+\delta)$,% RPA@PBE0	$\Delta E(-\delta)$, % RPA@PBE0	$\Delta E^{\mathrm{bind}}_{total}(+\delta)$,meV (PBE0)	$\Delta E^{\mathrm{bind}}_{total}(-\delta)$,meV (PBE0)
ammonia	-209	287	10	-3
cyclopentane	-49	18	177	129
neopentane	-48	20	82	70
ethyne	-62	62	17	14
benzene*	-12	13	59	14
N-methylacetamide*	-71	16	-31	34
pyrazine*	-43	33	30	21
pyridine*	-27	99	-44	-6
water	-1	3	116	119

Supplementary Table 2. FID of amino acid dimers at equilibrium (in meV) obtained from numerical RPA correlations, analytic estimation, RPA total binding energy and averaged FID for positive and negative charges: $W_c^{\text{bind}}(\delta) = \frac{1}{2}\left[E_c^{\text{bind}}(-\delta) - E_c^{\text{bind}}(\delta)\right]$ calculated on PBE0 orbitals.

system	RPA correlation($+\delta$)	RPA correlation($-\delta$)	FID analytic($+\delta$)	FID analytic($-\delta$)	RPA total binding	RPA W_c^{bind}	W_c analytic
alanine	-39	30	-29	24	253	34	27
asparagine	-44	32	-40	36	973	38	38
cysteine	-39	28	-27	25	727	34	26
glutamine	-59	31	-60	52	951	45	56
isoleucine	-93	38	-50	41	868	66	46
leucine	-76	21	-70	60	438	48	65
phenylalanine	-179	66	-95	89	479	122	92
serine	-28	11	-31	24	329	19	28
valine	-59	11	-40	34	420	35	37

Supplementary Table 3. FID of K^+ ion complexes at equilibrium (in meV) obtained from numerical RPA correlations, analytic formula, effect of the external charge on total RPA energy, same on total DFT energy and RPA total binding energy.

system	method	RPA correlation	FID analytic	effect of charge on RPA total	effect of charge on DFT total	RPA total binding
K1S3T	PBE	-93	-67	797	860	-1813
	PBE0	-79	-60	807	844	-1850
K2S2T (adjacent)	PBE	-93	-61	826	891	-1939
	PBE0	-81	-55	837	873	-1983
K2S2T(diagonal)	PBE	-83	-68	709	765	-1621
	PBE0	-70	-61	717	752	-1651
K3S1T	PBE	-85	-63	693	744	-1636
	PBE0	-71	-57	700	733	-1671
K4S	PBE	-92	-57	781	838	-1994
	PBE0	-80	-52	790	823	-2043
K4T	PBE	-92	-64	874	947	-1893
	PBE0	-81	-59	886	927	-1932

Supplementary Table 4.FID of Ba^2+ ion complexes at equilibrium (in meV) obtained from numerical RPA correlations, analytic formula, effect of the external charge on total RPA energy, same on total DFT energy and RPA total binding energy.

system	method	RPA correlation	FID analytic	effect of charge on RPA total	effect of charge on DFT total	RPA total binding
Ba1S3T	PBE	-147	-140	3967	4012	-213
	PBE0	-104	-120	3856	3906	-164
Ba2S2T(adjacent)	PBE	-100	-123	3833	3898	-207
	PBE0	-80	-105	3725	3784	-160
Ba2S2T (diagonal)	PBE	-139	-134	3903	3945	-221
	PBE0	-100	-115	3789	3837	-173
Ba3S1T	PBE	-163	-123	3752	3756	-212
	PBE0	-111	-105	3629	3664	-167
Ba4S	PBE	-122	-112	3642	3665	-223
	PBE0	-92	-96	3523	3565	-180
Ba4T	PBE	-21	-137	3966	4122	-177
	PBE0	-41	-121	3887	3978	- 236

Supplementary Table 5.FID of Na^+ ion complexes at equilibrium (in meV) obtained from numerical RPA correlations, analytic formula, effect of the external charge on total RPA energy, same on total DFT energy and RPA total binding energy.

system	method	RPA correlation	FID analytic	effect of charge on total RPA	effect of charge on total DFT	RPA total binding
Na1S3T	PBE	-98	-88	1359	1468	-1696
	PBE0	-100	-79	1369	1424	-1726
Na2S2T(adjacent)	PBE	-101	-87	1333	1438	-1749
	PBE0	-102	-78	1343	1396	-1782
Na2S2T(diagonal)	PBE	-102	-85	1322	1423	-1725
	PBE0	-102	-76	1331	1382	-1757
Na3S1T	PBE	-113	-83	1291	1382	-1778
	PBE0	-110	-74	1299	1345	-1812
Na4S	PBE	-106	-81	1301	1395	-1758
	PBE0	-104	-72	1310	1357	-1794
Na4T	PBE	-104	-85	1441	1556	-1626
	PBE0	-103	-77	1451	1509	-1652

SUPPLEMENTARY NOTE 1

In the following two sections we give an analytic derivation for the field–induced dispersion (FID) term, given by Equation(2) in the main text.

Unperturbed Quantum Drude Oscillator

The unperturbed QDO within the Born-Oppenheimer approximation is described by the following Hamiltonian: $H_0 = -\frac{\hbar^2}{2\mu}\nabla^2_{\mathbf{r}} + \frac{1}{2}\mu\omega^2(\mathbf{R_0} - \mathbf{r})^{\mathbf{2}}$, where $\mathbf{R_0}$ are the coordinates of the oscillation center and $\mathbf{r}$ is a vector designating the position of the Drude particle. The spectrum of H_0 is given by $E^{(kl)} = (3/2 + k + l)\hbar\omega$ and the eigenvectors are $\psi_{klm}(\mathbf{r}) = \mathcal{R}_{k,l}(r,\theta)\mathcal{Y}_{lm}(\theta,\phi)$, where $\{r,\theta,\phi\}$ are polar coordinates and $\{k,l,m\}$ being the radial and angular momentum quantum numbers. The wave function ψ_{klm} is composed by spherical harmonics $\mathcal{Y}_{lm}$ and $\mathcal{R}_{k,l} \simeq r^l\mathcal{L}_k^{l+1}\left(\frac{m\omega}{\hbar}r^2\right)$, where $\mathcal{L}_k^l$ are the generalized Laguerre polynomials.

Derivation of field-induced dispersion term

First three consecutive orders of the Rayleigh-Schrödinger perturbation theory, built on unperturbed eigenfunctions, are given by the well-known relations:

$$E^{(1)} = \langle 0| H' |0\rangle \,, \tag{1}$$

$$E^{(2)} = \sum_{k\neq 0} \frac{|\langle 0| H' |k\rangle|^2}{\left(E^{(0)} - E^{(k)}\right)}, \tag{2}$$

$$E^{(3)} = \sum_{k\neq 0, m\neq 0} \frac{\langle 0| H' |m\rangle \langle m| H' |k\rangle \langle k| H' |0\rangle}{\left(E^{(0)} - E^{(k)}\right)\left(E^{(0)} - E^{(m)}\right)} - \langle 0| H' |0\rangle \sum_{m\neq 0} \frac{|\langle 0| H' |m\rangle|^2}{\left(E^{(0)} - E^{(m)}\right)^2}, \tag{3}$$

where the perturbation includes the interaction between QDOs and influence of the external field $H' = H_A + H_B + H_{AB}$. Using addition theorem for spherical harmonics one can factorize the Coulomb potential and write the Hamiltonian of the interacting multipoles in the form: $H_{AB} = \sum_{l,l'=0}^{\infty}\sum_{m,m'} Q^A_{lm} T^{AB}_{lm;l'm'}(\mathbf{R}) Q^B_{l'm'}$, where $\mathbf{R}$ is a vector connecting the centers of multipoles, $Q^{A/B}_{lm}$ are complex multipole tensors in a spherical representation and defined in

the global coordinate system. The multipole interaction function takes the form [1–6]:

$$T_{l_A m_A l_B m_B}(\mathbf{R}) = (-1)^{l_A} \sqrt{\frac{(2l_A + 2l_B + 1)!}{(2l_A)!(2l_B)!}} \times \begin{pmatrix} l_A & l_B & l_A + l_B \\ m_A & m_B & -(m_A + m_B) \end{pmatrix} I_{l_A+l_B,-(m_A+m_B)}(\mathbf{R}), \tag{4}$$

where $I_{l,m}(\mathbf{R})$ are normalized irregular spherical harmonics and large brackets represent Wigner $3j$ symbol [7]. $T_{l_A m_A l_B m_B}(\mathbf{R})$ is a purely geometrical term that depends on orientation and distance between multipoles. Sometimes it is more convenient to use a real representation of Supplementary Equation (4) and Q_{lm} and to work in the local coordinate system. The relevant transformation and components of $T_{l_A m_A l_B m_B}$ are listed in [1–6].
The polarizabilities of QDO are defined by [1, 8]:

$$\alpha_{lm,l'm'} = \sum_n \frac{\langle 0| Q_{lm} |n\rangle \langle n| Q^\dagger_{l'm'} |0\rangle}{E_n - E_0} + \frac{\langle 0| Q^\dagger_{lm} |n\rangle \langle n| Q_{l'm'} |0\rangle}{E_n - E_0}, \tag{5}$$

where subscripts l, l' define the multipole order ($l = 1$ is a dipole, $l = 2$ is a quadrupole polarizability,...). In the isotropic case the polarizability is diagonal $\alpha_{lm,l'm'} = \alpha_l \delta_{l,l'} \delta_{m,m'}$. The ground state wavefunction of a QDO is given by $|0\rangle = \left(\frac{m\omega}{\pi}\right)^{3/4} \exp\left(-\frac{m\omega r^2}{2}\right)$, and the ground-state charge density has a Gaussian form of $n_0 = \frac{1}{(2\pi)^{3/2}\sigma^3} \exp\left(-\frac{r^2}{2\sigma^2}\right)$ and is given in terms of QDO broadening parameter $\sigma = \frac{1}{\sqrt{2m\omega}}$. This allows to build scaling relations between QDO volume $V = \sqrt{\frac{128}{\pi}}\sigma^3$ and polarizabilities $\alpha_1 \simeq V^{4/3}$, $\alpha_2 \simeq V^2$. These result in a Tkatchenko-Scheffler-like parametrisation of the effective quadrupole polarizability: $\alpha_2^{\text{eff}} = \alpha_2^{\text{free}} \left(\frac{V_h^{\text{eff}}}{V_h^{\text{free}}}\right)^2$, where α_2^{free} and V^{free} are the quadrupole polarizability and Hirshfeld volume of a free atom, respectively, and V_h^{eff} is an effective Hirshfeld volume, that accounts for the local chemical environment.
The terms in Supplementary Eqations (1)-(3) include electrostatic, induction and dispersion interactions. In order to describe long-range interactions, Ref.[8] developed a diagrammatic technique, which includes all orders of the polarization and dispersion interactions. The two first non-trivial terms in the perturbation theory caused by an external charge are associated with the two diagrams shown in Supplementary Figure 1.

Both of these terms have a closed loop-like structure and appear in the third order of the perturbation expansion. However, these diagrams have different physical origin. The

diagram on Supplementary Figure 1 (a) arises from the dispersion interaction between two QDOs, one being in a dipolar state, while bringing the other to the excited quadrupolar state via polarization induced by an external charge. Conversely, the term on Supplementary Figure 1 (b) forms a closed loop via the third block and represents a mixed term with electrostatic interaction. In order to obtain the analytical representation of these diagrams one should calculate Supplementary Equation (3), substitute H', and expand the matrix elements in the numerator. The polarization-dispersion term on Supplementary Figure 1 (a) arises from the matrix element proportional to $\langle 0| H_{AB} |m\rangle \langle m| H_A |k\rangle \langle k| H_{AB} |0\rangle$. We note that this term is not symmetric with respect to indices A and B, since it describes the QDO A quadrupolar state. However the total energy expansion term $E^{(3)}$ includes as well a symmetric term for the oscillator B. Matrix elements that appear in the expansion could be calculated explicitly:

$$\langle 0| Q_{lm} |k'l'm'\rangle = C_l \left(2l+1\right)^{-1/2} \delta_{0k}\delta_{l,l'}\delta_{m,-m'}, \tag{6}$$

$$\begin{aligned} \langle 0l'm'| Q_{lm} \left|\widetilde{0l\widetilde{m}}\right\rangle &= \sqrt{\frac{4\pi}{2l+1}} \int_\Omega (-1)^{m'} \mathcal{Y}_{l',-m'} \mathcal{Y}_{l,m} \mathcal{Y}_{\widetilde{l}\widetilde{m}} d\Omega \int_r r^l \mathcal{R}_{0l'} \mathcal{R}_{0\widetilde{l}} dr = \\ &= \sqrt{(2l'+1)(2\widetilde{l}+1)} \left((-1)^{m'} \begin{pmatrix} l' & l & \widetilde{l} \\ 0 & 0 & 0 \end{pmatrix} \begin{pmatrix} l' & l & \widetilde{l} \\ -m' & m & \widetilde{m} \end{pmatrix} \right) \frac{C_{l+\widetilde{l}}}{C_{\widetilde{l}}} \delta_{l',l+\widetilde{l}} \end{aligned}, \tag{7}$$

where $C_l = (2l+1)!! \left(\frac{\hbar}{2\mu\omega}\right)^l$. During the derivation we use the orthogonal relation between spherical harmonics $\mathcal{Y}_{l,m}$ and $3j$-symbol notation. Substituting (6),(7) in Supplementary Equation 3 one gets a polarization-dispersion correction for the QDO A:

$$E^A_{\mathrm{FID}} = -3\delta \sum_{\substack{l'_A,m_A,m_A, \\ l_B,m_B, \\ \widetilde{l_A},\widetilde{m_A}}} \frac{T^{AB}_{l'_A+\widetilde{l_A},-m_A;l_B,-m_B} T^{AB}_{\widetilde{l_A},m_A;l_B,m_B}}{\left(\left(l'_A+\widetilde{l_A}\right)\hbar\omega_A + l_B\hbar\omega_B\right)\left(\widetilde{l_A}\hbar\omega_A + l_B\hbar\omega_B\right)} \times$$
$$\frac{C^2_{l_B}}{2l_B+1} \frac{C^2_{l'_A+\widetilde{l_A}}}{2(l'_A+\widetilde{l_A})+1} \frac{(2(l'_A+\widetilde{l_A})+1)}{\widetilde{R}_A^{l'_A+1}} \left((-1)^{m_A} \begin{pmatrix} l'_A+\widetilde{l_A} & l'_A & \widetilde{l_A} \\ 0 & 0 & 0 \end{pmatrix} \begin{pmatrix} l'_A+\widetilde{l_A} & l'_A & \widetilde{l_A} \\ -m_A & m'_A & \widetilde{m_A} \end{pmatrix} \right), \tag{8}$$

Supplementary Equation (8) implies the selection rules for angular momentum: $l' = l + \widetilde{l}$ and $m' = m + \widetilde{m}$. Applying the following relation between isotropic polarizabilities $\frac{C_l^2}{2l+1}\delta_{ll'}\delta_{mm'} = \frac{l\hbar\omega}{2q^2}\alpha_{lm;l'm'}$ one gets the lowest order ($l'_A = \widetilde{l_A} = 1$) dipole-quadrupole dispersion term:

$$E^A_{\rm FID} = \frac{\delta}{2}\frac{\alpha^B_1\alpha^A_2\omega_A\omega_B}{(2\omega_A+\omega_B)(\omega_A+\omega_B)}\frac{1}{\widetilde{R}^2_A}\sum_{m_A,m_B} T_{2,-m_A;1,-m_B}T_{1,m_A;1,m_B}\sqrt{4-m^2_A}. \tag{9}$$

The other bubble-like electrostatic term, that appears in third-order perturbation theory originates from $\langle 0|\, H_A\, |m\rangle \langle m|\, H_{AB}\, |k\rangle \langle k|\, H_B\, |0\rangle$ matrix element and is given by the sum:

$$E_{\rm ind} = \frac{3}{2}\delta^2 \sum_{\substack{l_A,m_A\\ l_B,m_B}} T^{AB}_{l_A,m_A;l_B,m_B}\frac{\alpha^A_{l_A}\alpha^B_{l_B}}{\widetilde{R}^{l_A+1}_A\widetilde{R}^{l_B+1}_B}, \tag{10}$$

which starts from $l_A = 2, l_B = 1$ or $l_A = 1, l_B = 2$, since the dipole interaction function is traceless. At lowest order $E_{\rm ind}$ takes the form:

$$E_{\rm ind} = \frac{3}{2}\delta^2\left[\frac{\alpha^A_2\alpha^B_1}{\widetilde{R}^3_A\widetilde{R}^2_B}\sum_{m_A,m_B} T^{AB}_{2,m_A;1,m_B} + \frac{\alpha^B_2\alpha^A_1}{\widetilde{R}^3_B\widetilde{R}^2_A}\sum_{m_A,m_B} T^{AB}_{1,m_A;2,m_B}\right], \tag{11}$$

which scales as R^{-4} with distance between QDOs and is proportional to δ^2 .

Tightly converged RPA (and MP2) calculations

In this section we give technical details behind RPA calculations, that are used as a reference for FID benchmark in the main text. Similar technical settings were employed for MP2 calculations. RPA calculations [9, 10], done with FHI-aims code [11], serve as an accurate reference for comparison with analytic FID results. RPA approach includes proper accounting of a long-range correlation energy contributions and coupling of a static charge to these correlations, since it accounts for electrodynamic response screening up to infinite order. The following numerical results are obtained using following exchange-correlation functional: PBE [12], Hartree-Fock (HF) and hybrid PBE0 [13, 14] with atom-centered orbital(NAO) basis sets with valence-correlation consistency (VCC), designed to contain explicit sums over unoccupied states [15] within full-potential all-electron approach. The basis set incompleteness error was reduced by the two-point extrapolation scheme applied to the basis set sequence NAO-VCC-4Z and NAO-VCC-5Z [15]:

$$E(\infty) = \frac{E(n_1)n^3_1 - E(n_2)n^3_2}{n^3_1 - n^3_2}, \tag{12}$$

where $n_{1,2}$ - are indexes of NAO-VCC-nZ basis sets, being equal to 4 and 5 in this work.

FID contribution to the total binding energy of molecular dimers

This section contains supplementary information on the total binding energies calculated with RPA@PBE0 for the small molecular dimers from the Table 1 of the main text and corresponding PBE0 contributions.

We also use the following ratio, that shows how the rate of FID–(DQ) binding energy contribution to the total binding energy:

$$\Delta E = \frac{E_c^{\text{bind}}(\delta \neq 0) - E_c^{\text{bind}}(\delta = 0)}{E_{\text{tot}}^{\text{bind}}(\delta \neq 0) - E_{\text{tot}}^{\text{bind}}(\delta = 0)}. \tag{13}$$

In Eq.13 components of the total binding energies are calculated with RPA@PBE method and are given by $E_{\text{tot}}^{\text{RPA}} = E_{\text{tot}}^{\text{DFT}} - E_{\text{xc}}^{\text{DFT}} + E_{\text{x}}^{\text{EX}} + E_{\text{c}}^{\text{RPA}}$, where $E_{\text{tot}}^{\text{DFT}}$ is a DFT total energy, obtained from PBE self-consistent calculations, $E_{\text{xc}}^{\text{DFT}}$ – is a DFT exchange-correlation contribution,E_{x}^{EX} – exact exchange and $E_{\text{c}}^{\text{RPA}}$ is a non-local RPA correlation term [9, 10].

Damping function

In this section we give details behind FID calculations in ionic channel and amino acid dimers. Since amino acids have relatively close-packed configuration around the ion, a damping function should be introduced to attenuate the long-range FID at short distances. For this purpose we chose the Fermi-type damping function, parametrized similarly to TS method [16], parametrized for PBE ($d = 20$, $s_r = 0.94$) and PBE0 ($d = 20$, $s_r = 0.96$) functional within S22 dataset:

$$f_{damp}(R) = \frac{1}{1 + \exp\Big\{ \Big[-d\big(\frac{R}{s_R(R_A^0 + R_B^0)} - 1\big) \Big] \Big\}} \tag{14}$$

FID term is the following:

$$\begin{aligned} E_{\text{disp}}^{(3)A} &= -\frac{\delta}{2} \frac{\alpha_1^B \alpha_2^A \omega_A \omega_B}{(2\omega_A + \omega_B)(\omega_A + \omega_B)} \frac{1}{\widetilde{R}_A^2} \times \\ &\sum_{m_A, m_B} T_{2,-m_A;1,-m_B} T_{1,m_A;1,m_B} \sqrt{4 - m_A^2} \end{aligned} . \tag{15}$$

The fitting parameters s_R and d were kept the same as in the TS method. Now we sum up intermolecular FID terms in a pairwise manner over atom pairs, belonging to different

molecules, with a damping function as a prefactor:

$$E = \sum_{A,B} f_{damp}(R) \left(E_{\rm disp}^{(3)A}(R, \widetilde{R}) + E_{\rm disp}^{(3)B}(R, \widetilde{R}) \right) \tag{16}$$

$E_{\rm disp}^{(3)A/B}(R)$ corresponds to molecule A or B being excited to quadrupolar state.

FID of ion complexes

Below we give detailed information on RPA and DFT energies for the set of ions K^+, Na^+, Ba^{2+}, shown on Fig. 4 of the main text. The T $\rightarrow$ S substitutions result in minor topology changes, therefore in our calculations we used geometries reported in Ref. [17], modelling the ion with a point charge.

The relative contribution of FID to the dispersion energy ranges from 10% in case of K^+ to 30% for Ba^2+ complex (see Supplementary Figure 2 (a), where FID is compared to RPA@PBE0 and MBD binding energies). The effect of the charge on the binding energy is even more pronounced in substitutional energies (see Supplementary Figure 2 (b)-(d)) and reaches a maximum of 35 meV for Ba^{2+} ion system.

SUPPLEMENTARY REFERENCES

1. Stone, A. J. *The Theory of Intermolecular Forces* (Oxford University Press, 2013).
2. Popelier, P. L. A., Joubert, L. & Kosov, D. S. Convergence of the electrostatic interaction based on topological atoms. *J. Phys. Chem. A* **105**, 8254–8261 (2001).
3. Panas, I. Practical expressions for the two-center multipole expansion of r121. *International Journal of Quantum Chemistry* **53**, 255–263 (1995).
4. Hättig, C. & Heß, B. A. Calculation of orientation-dependent double-tensor moments for Coulomb-type intermolecular interactions. *Mol. Phys.* **81**, 813–824 (1994).
5. Price, S., Stone, A. & Alderton, M. Explicit formulae for the electrostatic energy, forces and torques between a pair of molecules of arbitrary symmetry. *Mol. Phys.* **52**, 987–1001 (1984).
6. Hättig, C. Recurrence relations for the direct calculation of spherical multipole interaction tensors and Coulomb-type interaction energies. *Chem. Phys. Lett.* **260**, 341 – 351 (1996).
7. Abramowitz, M. & Stegun, I. *Handbook of Mathematical Functions* (Dover Publications, 1965).
8. Jones, A. P., Crain, J., Sokhan, V. P., Whitfield, T. W. & Martyna, G. J. Quantum Drude oscillator model of atoms and molecules: Many-body polarization and dispersion interactions for atomistic simulation. *Phys. Rev. B* **87**, 144103 (2013).
9. Ren, X. *et al.* Resolution-of-identity approach to Hartree-Fock, hybrid density functionals, RPA, MP2 and GW with numeric atom-centered orbital basis functions. *New J. Phys.* **14**, 053020 (2012).
10. Ren, X., Rinke, P., Joas, C. & Scheffler, M. Random-phase approximation and its applications in computational chemistry and materials science. *J. Mater. Sci.* **47**, 7447–7471 (2012).
11. Blum, V. *et al.* Ab initio molecular simulations with numeric atom-centered orbitals. *Comput. Phys. Commun.* **180**, 2175 – 2196 (2009).
12. Perdew, J. P., Burke, K. & Ernzerhof, M. Generalized gradient approximation made simple. *Phys. Rev. Lett.* **77**, 3865–3868 (1996).
13. Ernzerhof, M. & Scuseria, G. E. Assessment of the Perdew-Burke-Ernzerhof exchange-correlation functional. *J. Chem. Phys.* **110**, 5029–5036 (1999).

14. Adamo, C. & Barone, V. Toward reliable density functional methods without adjustable parameters: The PBE0 model. *J. Chem. Phys.* **110**, 6158–6170 (1999).

15. Zhang, I. Y., Ren, X., Rinke, P., Blum, V. & Scheffler, M. Numeric atom-centered-orbital basis sets with valence-correlation consistency from H to Ar. *New J. Phys.* **15**, 123033 (2013).

16. Tkatchenko, A. & Scheffler, M. Accurate molecular van der Waals interactions from ground-state electron density and free-atom reference data. *Phys. Rev. Lett.* **102**, 073005 (2009).

17. Rossi, M., Tkatchenko, A., Rempe, S. B. & Varma, S. Role of methyl-induced polarization in ion binding. *Proc. Natl. Acad. Sci.* **110**, 12978–12983 (2013).
